# Very Low–Carbohydrate Breakfast Intervention for Adults with Type 2 Diabetes and Persistent Hyperglycemia: Protocol for a Digital, Nonrandomized Pre-Post Study

**DOI:** 10.2196/81041

**Published:** 2026-01-28

**Authors:** Annika Hansen, Kaitlyn Raymond, Alexander Simon, Sarah Kim, Hovig Bayandorian, Deanna Marriott, Laura R. Saslow

**Affiliations:** 1 School of Medicine University of Utah Salt Lake City, UT United States; 2 School of Nursing University of Michigan Ann Arbor, MI United States; 3 School of Medicine University of California, San Francisco San Francisco, CA United States

**Keywords:** type 2 diabetes, glycemic control, very low-carbohydrate breakfast, nutrition, glycemic variability, lifestyle intervention

## Abstract

**Background:**

More than 15% of US adults with type 2 diabetes have persistent hyperglycemia. Adults with persistent hyperglycemia and type 2 diabetes have an elevated health risk of a variety of outcomes, including amputation and mortality from cardiovascular disease and from all causes. Nutrition-focused interventions can be effective for improving glycemic control, reducing antihyperglycemic medications, and reducing body weight, all of which are critical outcomes for adults with type 2 diabetes. Carbohydrate intake impacts postprandial glycemia more than any other dietary factor. The American Diabetes Association now recommends a very low–carbohydrate diet, because of its ability to improve glycemic control, for the treatment of type 2 diabetes. However, typical nutrition-focused interventions can be burdensome, as the interventions often have complex instructions and require changing one’s diet completely. Additionally, adults with type 2 diabetes and persistent hyperglycemia may be more likely to have low health literacy levels, which can be a barrier to adherence to complex interventions.

**Objective:**

This study aimed to evaluate the effectiveness of a digital, small-steps intervention that focuses on implementing a very low–carbohydrate dietary pattern specifically at breakfast for adults with type 2 diabetes and persistent hyperglycemia. The goal is to determine whether this targeted dietary modification can lead to reductions in hemoglobin A_1c_ levels and decreased use of antihyperglycemic medications, without requiring participants to change their entire diet.

**Methods:**

The Breakfast Study will enroll adults with a hemoglobin A_1c_ (HbA_1c_) of 7.0% or higher to our online, 4-month intervention, which will teach participants to change their breakfasts to be very low in carbohydrates. We will measure acceptability and feasibility, plus critical efficacy outcomes, such as changes in HbA_1c_, antihyperglycemic medications, glycemic variability, body weight, blood pressure, and lipids. We will also test whether factors such as sex and baseline insulin resistance significantly moderate the impact of the intervention on change in HbA_1c_ and antihyperglycemic medications. If the results are promising, we will conduct a follow-up, powered, longer randomized controlled trial of this approach. As the prevalence of type 2 diabetes and the understanding of personalized interventions continue to increase, there is a critical need to provide additional effective options for population-level type 2 diabetes treatment strategies, especially for adults with type 2 diabetes and persistent hyperglycemia.

**Results:**

As of October 2025, we have enrolled 119 participants. The results will be published separately.

**Conclusions:**

The Breakfast Study is a nonrandomized, pre-post trial to assess the acceptability, feasibility, and preliminary effectiveness of an accessible, very low–carbohydrate breakfast for adults with type 2 diabetes and persistent hyperglycemia. This study could provide support for continued research investigating how to lower barriers to dietary interventions for type 2 diabetes.

**Trial Registration:**

ClinicalTrials.gov NCT05986097; https://clinicaltrials.gov/study/NCT05986097

**International Registered Report Identifier (IRRID):**

DERR1-10.2196/81041

## Introduction

### Background and Rationale

Type 2 diabetes is one of the largest and most prevalent contemporary public health problems in the United States. If the current trajectory of prevalence continues, 1 in 3 adults in the United States will have type 2 diabetes by 2050 [1]. It is the most expensive health condition in terms of total health care spending nationwide [2], and these costs have continued to grow, from roughly US $175 billion in 2007 to US $327 billion in 2017 [3,4].

More than 15% of US adults with type 2 diabetes have persistent hyperglycemia [5]. Patients with type 2 diabetes have an elevated risk of a variety of health outcomes, including stroke, blindness, renal failure, premature death, and mortality from cardiovascular disease and all causes [6]. More than half of all amputations in the United States are due to complications from type 2 diabetes [7].

Nutrition-focused interventions may be effective for improving critical outcomes for adults with type 2 diabetes, such as improving glycemic control, reducing antihyperglycemic medications, and reducing body weight. Carbohydrate intake has the strongest impact on postprandial glycemia of any dietary factor. A very low–carbohydrate diet, because of its ability to improve glycemic control and reduce antihyperglycemic medications, is now recommended by the American Diabetes Association for the treatment of type 2 diabetes [8]. However, typical nutrition-focused interventions can be burdensome, as they often use complex instructions and require a complete revision of one’s diet. Additionally, adults with type 2 diabetes and persistent hyperglycemia are more likely to have low health literacy levels, which can be a barrier to adherence to complex self-management interventions [9,10].

The benefits of a very low-carbohydrate diet may be available to individuals who change only their breakfasts to be very low in carbohydrates, rather than modifying their entire diet. For example, in a 3-month randomized trial of 59 adults with overweight or obesity with type 2 diabetes, there was a greater reduction in hemoglobin A_1c_ (HbA_1c_) and antihyperglycemic medications in those assigned to eat a higher-calorie, reduced-carbohydrate breakfast compared to those assigned to eat a smaller, higher-carbohydrate breakfast. However, the participants already had well-controlled type 2 diabetes (average baseline HbA_1c_ level was 6.9%) and the reduced carbohydrate breakfast still had a moderate amount of carbohydrates (39% of calories from carbohydrates) and therefore would not be considered a very low–carbohydrate meal [11].

In a feeding trial of 23 adults with type 2 diabetes, participants consumed isocaloric diets that differed only in whether breakfast was very low in carbohydrates or higher in carbohydrates. On days that the participants consumed the very low–carbohydrate diet breakfast, their morning postprandial hyperglycemia was reduced, and their before-dinner perception of hunger was lower [12].

A different large trial found that postprandial glucose variability in response to a standardized breakfast predicts subsequent hunger and, thus, breakfast may be an important target for changing the day-long eating behavior. Researchers tracked the continuous glucose responses of more than 1000 people and found a correlation between postprandial glucose levels and subsequent hunger and calorie consumption. Researchers found that larger blood glucose dips after eating led to greater later-day calorie consumption. This suggests that, by eating meals that reduce postprandial glycemic variability, subsequent hunger and, therefore, subsequent eating might be reduced [13]. This trial was conducted in people without type 2 diabetes. Previous research shows that people with type 2 diabetes have, on average, twice as large a blood glucose spike in response to eating carbohydrates as compared to people without type 2 diabetes [14]. This suggests that reducing carbohydrate consumption for glycemic control in people with type 2 diabetes is of even greater importance.

A 3-month randomized trial investigated the effects of a low-carbohydrate breakfast versus a low-fat breakfast on glycemic control in individuals with type 2 diabetes. The low-carbohydrate diet showed a reduction in HbA_1c_ levels. Continuous glucose monitoring revealed improved metrics for the low-carbohydrate group, including lower mean and maximum glucose levels, reduced glycemic variability, and increased time within the target glucose range. However, this study only included participants who had an HbA_1c_ level of 6.5% to 8.5% and not those with persistent hyperglycemia [15].

The above research suggests the benefit of a very low–carbohydrate diet breakfast for improving glycemic control, reducing the need for antihyperglycemic medications, reducing postprandial glucose variability, and reducing both subsequent hunger and calorie consumption.

### Objectives

To conduct an acceptability, feasibility, and preliminary effectiveness trial in 119 adults with type 2 diabetes and persistent hyperglycemia. We will assign adults with an HbA_1c_ of 7.0% or higher to our 4-month, digital, small-steps, very low-carbohydrate breakfast–focused program (The Breakfast Study).

We will examine the acceptability and feasibility of the very low-carbohydrate breakfast intervention, including participants’ satisfaction with the intervention and their diabetes treatment satisfaction, health-related quality of life, and dietary adherence. For feasibility, we will record the mean number of completed sessions (out of 16 possible). We have denoted a 50% attendance of assigned sessions as our a priori benchmark for feasibility. Using an SD of 30%, this trial has >85% power to detect this level of completion (vs 50% completion) using a 2-tailed *t* test at an ⍺ level of 5%.

We will assess health and psychological outcomes, including HbA_1c_, antihyperglycemic medications, glycemic variability, body weight, lipids, insulin resistance, blood pressure, self-efficacy for dietary adherence, and satiety.

We will test whether factors such as sex, emotional eating, and baseline insulin resistance significantly moderate the impact of the intervention on change in HbA_1c_.

An effective and simple intervention for adults with diabetes and persistent hyperglycemia is needed to reduce both HbA_1c_ levels and antihyperglycemic medications. We hypothesize that a digital, small-steps, very low-carbohydrate breakfast–focused program for adults with type 2 diabetes and persistent hyperglycemia could be effective at achieving these aims. Our primary aim is to assess the acceptability and feasibility of such a study.

## Methods

### Trial Design

This online, remote trial enrolls adults with an HbA_1c_ of 7.0% or higher and assigns them to a 4-month intervention focused on teaching participants to adopt very low–carbohydrate breakfasts.

### Study Setting

The intervention will be delivered remotely and is technology supported. Participants can live anywhere in the United States.

### Eligibility Criteria

Key inclusion criteria include (1) a current HbA_1c_ level of >7.0%, (2) the ability to read and speak English, (3) age 18-80 years, and (4) the willingness to regularly check blood glucose levels.

Exclusion criteria include (1) unable to provide informed consent; (2) pregnant or planning to become pregnant in the next 12 months, or breastfeeding or less than 6 months post partum; (3) vegan; (4) a C-peptide test result that suggests type 1 diabetes, a previous diagnosis of type 1 diabetes, or latent autoimmune diabetes of adults (if C-peptide is <0.75 ng/mL, participants are ineligible; if C-peptide is between 0.75 ng/mL and 1.5 ng/mL, these participants will need to get an additional test, Glutamic Acid Decarboxylase 65, to rule out latent autoimmune diabetes of adults; if the Glutamic Acid Decarboxylase 65 test result is ≥5 U/mL, participants will be ineligible); (5) cancer; (6) heart failure; (7) kidney failure; (8) liver failure; (9) an untreated mental health condition; (10) currently following a very low–carbohydrate diet or breakfasts; (11) abnormal thyroid levels, if baseline thyroid stimulating hormone is out of range, a free T3 or free T4 test may be ordered; (12) triglyceride >999 mg/dL; (13) a change in diabetes medication within the last 3 months; (14) alcohol use disorder; (15) previous bariatric surgery; (16) difficulty chewing or swallowing; (17) dependence on others for food preparation; and (18) currently enrolled in another investigative study that might conflict with this research.

Participants with a triglyceride level reported on the initial blood draw of 300-499 mg/dL will be enrolled with the requirement of a follow-up lipids test 2 weeks into the intervention. For triglyceride levels 500-999 mg/dL, we will have the participant first discuss the result with their primary care physician so that any medication or dietary changes can occur before starting the study. If any significant changes were made by the participant’s physician (eg, addition or removal of a medication), we would measure a new baseline at the appropriate time. If triglycerides are greater than 999 mg/dL, the participant will not be enrolled. Such tests may also be ordered after an initial meeting with the study physician, based on clinical judgment.

### Who Will Take Informed Consent?

An authorized team member, who has undergone the necessary ethical training and received clearance from the institutional review board, will discuss the details of the study and consent forms, as well as respond to any questions. It will be emphasized that participation in the study is completely optional.

### Intervention

#### Intervention Description

**Figure 1:**
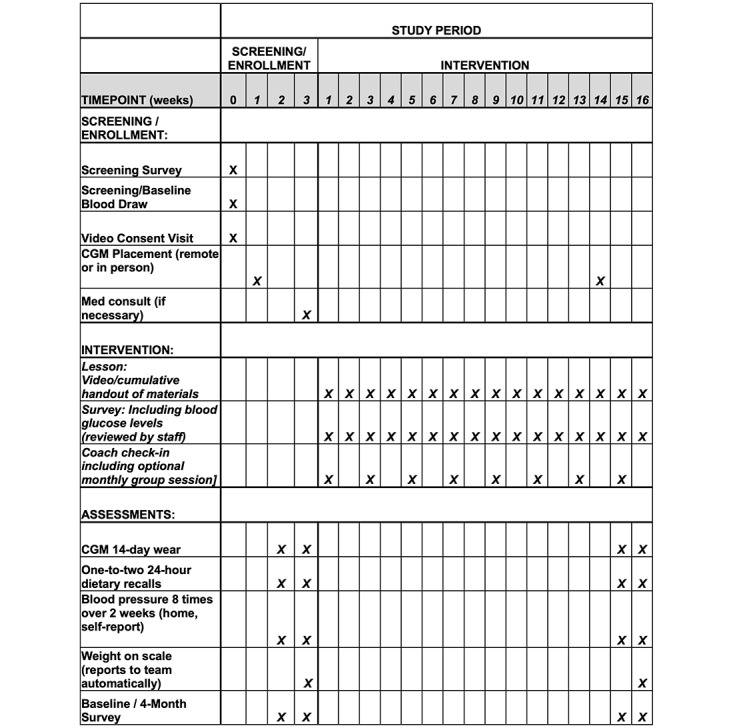
Timeline of planned contact for enrollment, study intervention, and assessments for pre-post program for adults with type 2 diabetes. CGM: continuous glucose monitor.

We propose to enroll 84 adults, after attrition, with type 2 diabetes and persistent hyperglycemia, in our 4-month online intervention. Assessments will take place at baseline and 4 months later, at the end of the intervention. Participants will complete an online course for a very low–carbohydrate breakfast diet program. This online program allows us to conduct the intervention remotely. Participants will receive weekly classes online, a food guide and recipe booklet through the mail, and a diet coach to support them. For details, see the SPIRIT (Standard Protocol Items: Recommendations for Interventional Trials) flowchart in Figure 1 for a schedule of the intervention timeline (checklist in Multimedia Appendix 1).

#### Very Low–Carbohydrate Breakfasts

Study materials will encourage eating a very low–carbohydrate breakfast each day. The very low–carbohydrate diet intervention will focus on food choices, without calorie counting or calorie restriction. We will recommend that breakfast meals should have no more than 10 nonfiber grams of carbohydrates or no more than 20% of total calories from carbohydrates. To avoid confusion about how to interpret this, we will provide participants with copious food options that will vary in their complexity and cost. For examples, see Textbox 1. Through coaching, we will help participants to find options that they enjoy and find accessible.

**Examples of very low–carbohydrate diet breakfast options as part of a pre-post 4-month program for adults with type 2 diabetes.**

**Breakfast options**

- Classic eggs, bacon
- Sausage breakfast casserole
- Low-carbohydrate pancakes and waffles
- Cottage cheese with fruit and nuts
- Almond and coconut crunch cereal
- Vanilla pecan bars
- Cheesy breakfast pizza
- Low-carbohydrate English muffin in a mug

#### Criteria for Discontinuing or Modifying Allocated Interventions

In the event of any serious adverse events resulting from the intervention, participants will immediately stop the diet and will be included in the intention-to-treat analysis. If there are any changes to the participants’ medical treatment plan that conflict with the intervention, participants will also stop the intervention. Furthermore, participants will be notified that they have the right to retract their consent to participate at any time and may refuse to answer any questions from the study team.

#### Strategies to Improve Adherence to Interventions

To improve adherence to the intervention, there will be responsive coaching (via phone, video, and email) so that participants’ questions and comments are replied to in a timely and supportive manner. Reminders about targeted behaviors are tied to greater adherence, so participants will be sent text messages to provide feedback, as well as to motivate, educate, and remind them about targeted behaviors and skills about 5 times per week. Monthly group video sessions will also be offered as an optional way for participants to receive support.

#### Relevant Concomitant Care Permitted or Prohibited During the Trial

Relevant concomitant care is permitted during the trial.

#### Provisions for Posttrial Care

We will not be providing posttrial care. We do not anticipate harm due to trial participation.

### Outcomes

#### Aim 1: Assess Acceptability and Feasibility—Acceptability: Intervention Satisfaction

At month 4, we will ask participants, “How would you rate your overall satisfaction with the program?” on a 0-6 Likert scale (0=very dissatisfied; 6=very satisfied).

Additionally, each week, participants will receive a check-in survey. The survey will inquire (1) how often they ate a very low–carbohydrate breakfast on a scale from 1 to 7 and how much they enjoyed eating a very low–carbohydrate breakfast on a scale from 1 to 7 (1=not at all to 7=very much so), (2) what their highest and lowest blood sugar readings were over the past week and what were the circumstances of the readings (only asked of those taking insulin, sulfonylureas, or meglitinides), and (3) any medication changes they may have made over the past 7 days.

#### Other Exploratory Outcomes for Acceptability

##### Diabetes Treatment Satisfaction

The Diabetes Treatment Satisfaction Questionnaire, an 8-item scale, measures 2 factors: treatment satisfaction (along dimensions such as flexibility and convenience) and burden from hyperglycemia and hypoglycemia. It is a short, easy-to-answer scale with low participant burden. We will use the Diabetes Treatment Satisfaction Questionnaire at baseline and 4 months later [16].

##### Health-Related Quality of Life

Health-related quality of life is a central aspect of well-being. We will use the Patient-Reported Outcomes Measurement Information System 29 at baseline and 4 months later [17]. Patient-Reported Outcomes Measurement Information System 29 is a questionnaire [17] that assesses physical functioning, anxiety, depression, fatigue, sleep disturbance, social functioning, and pain.

##### Physical Symptoms

At baseline and 4 months later, we will also measure physical symptoms using a short, face-valid measure of physical symptoms [18]. Participants report how often over the past month they have had symptoms such as dizziness, diarrhea, constipation, headaches, blurred vision, nausea, pain in their hands or feet, heartburn, and acne.

##### Exploratory Open-Ended Survey Questions and Qualitative Interviews

In the 4-month survey, all participants will be asked open-ended questions about the aspects of the program participants enjoyed, which aspects could be improved, what made it easier to adhere to the program, and which factors made adherence more difficult.

To better inform the next phase of our study, we will conduct qualitative semistructured interviews at the end of the trial. This will allow us to explore the experience of the participants in greater depth, including their perceptions of the intervention, as well as barriers to and facilitators of their ability to make long-lasting dietary changes. We will model the interview guide and data analysis on prior research, using a semistructured interview guide that includes open-ended questions to encourage participants’ perspectives, thoughts, and beliefs about the intervention and its components. Participants will be interviewed by nonintervention staff from our research team. These interviews will last approximately 20-30 minutes. We will create a summary of the qualitative data, with evidence from participant quotes, to explore participant experiences.

#### Other Exploratory Outcomes for Feasibility

##### Participant Recruitment and Progress Through the Trial

As recommended by the CONSORT (Consolidated Standards of Reporting Trials) guidelines, we will report on the percentage enrolled from each recruitment method, the number excluded and why, the number who chose not to enter the trial and why, and the number and reasons for withdrawal [19].

##### Dietary Adherence

We will assess dietary adherence with 1-2 unannounced 24-hour dietary recalls at baseline and at 4 months, conducted by a dietician. At the 4-month outcome mark, this will serve as a check on our intervention fidelity in terms of whether we have achieved our targeted macronutrient levels for breakfasts. We will explore what percentage of calories in participants’ breakfasts were derived from carbohydrates, as well as what percentage of participants ate breakfasts that had 20% or less of their calories from carbohydrates. We will also examine the macronutrient content and quality of other eating occasions throughout the day to assess whether breakfast quality impacted other eating patterns.

#### Aim 2: Assess Changes in Health and Psychological Outcomes

As appropriate, for the following measures, we will explore how each of these outcomes changed from the baseline to the end of the study. All blood tests will be done at Labcorp lab locations, with the rare exception that if participants are unable to go to a Labcorp location, we will mail them a DTI Laboratories, Inc home HbA_1c_ kit for the 4-month outcome.

##### HbA_1c_

HbA_1c_ is a standard measure of overall glycemic control in the clinical care of type 2 diabetes. A higher HbA_1c_ is associated with increased risk of microvascular complications from diabetes [20].

##### Antihyperglycemic Medications

The study team will ask participants about their current diabetes-related medication regimen using questions that have successfully been used in other studies [21]. We will use a medication-effect score, which combines dosage and strength of medications to assess the overall intensity of an antihyperglycemic medication regimen [22].

##### Diabetes Control

We will explore metrics of diabetes control and report on the percentage of participants who (1) lower their HbA_1c_ levels to below 7.0% without increasing their medication effect score, (2) lower their HbA_1c_ levels to below 6.5% without increasing their medication effect score, and (3) lower their HbA_1c_ levels to below 6.5%, without increasing their medication effect score, and take no antihyperglycemic medications other than metformin.

##### Glycemic Variability

Data will be analyzed from a continuous glucose monitor at baseline and 4 months. The research team will download sensor data at the end of the measurement period. We will assess the glucose variability and the proportion of time spent in the euglycemic (3-7.8 mmol/L) and hyperglycemic (≥ 11.1 mmol/L) states, following previous standards for interstitial glucose concentrations [23-25].

##### Body Weight

We will give participants a body weight scale and ask them to weigh themselves weekly in order to help tailor coaching support. However, our outcome of interest will be weight change from baseline to 4 months. We will examine average weight change and the percentage of participants who lose 5% of their body weight, a clinically meaningful amount of weight loss.

##### Lipids

At baseline and at 4 months, we will measure triglycerides and fractionated cholesterol using Labcorp’s Nuclear Magnetic Resonance LipoProfile [26]. This advanced lipid assay provides measurements that are better associated with elevated cardiovascular risk than conventional lipid assays [27].

##### Insulin Resistance

At baseline and 4 months, fasting insulin and glucose will be used to estimate insulin resistance by calculating Homeostatic Model Assessment-Insulin Resistance. Homeostatic Model Assessment-Insulin Resistance uses a single fasting blood draw to estimate insulin resistance [28].

##### Inflammation

At baseline and 4 months, high-sensitivity C-reactive protein, which is an acute phase reactant, will be assessed via blood draw.

##### Blood Pressure

We will mail participants a blood pressure cuff with instructions from the American Heart Association to correctly measure blood pressure. To control for the white-coat effect, we will use a home-based measurement [29]. We will ask participants to measure their blood pressure level 8 times during the 2 weeks before the study begins and the last 2 weeks of the study. We will use the average over those 2-week periods as our assessment of interest, with the change from baseline to 4 months being our outcome of interest.

##### Self-Efficacy for Dietary Adherence

At baseline and 4 months later, we will measure this with the 8-item Weight Efficacy Lifestyle Questionnaire Short-Form, which assesses self-efficacy for dietary adherence when facing a variety of challenging situations, including poor mood and social situations [30].

##### Satiety and Food Responsiveness

At baseline and 4 months later, we will assess whether the intervention improves these 2 factors using two 4-item subscales of the Adult Eating Behavior Questionnaire, one for each factor [31].

### Participant Timeline

Potential participants will make initial contact with the study by visiting the study website or reaching out to the study team through a dedicated study phone number or email address. Participants will be directed to a study website where they will take an initial screening survey. If participants appear initially eligible, the participant is invited to watch an online video describing the study and answer questions about their goals for participation and the pros and cons of participation (which previous research suggests can improve trial retention) [32]. Following the online video, they fill out the blood draw consent form. Once consented, participants will go for a screening and baseline fasting blood draw to assess parameters required for inclusion.

If potential participants remain eligible after the blood draw, our study staff will consent them on the phone or via a video meeting and review the study schedules, procedures, and assessments. We will send a fax to the primary care provider explaining the study and that the participant is eligible unless the physician objects. If we are not contacted by the physician in 2 weeks, we assume the physician is supportive. Participants will then (1) complete a baseline survey online, (2) participate in 1-2 unannounced 24-hour dietary recalls over the phone, and (3) wear a continuous glucose monitor mailed to them, or attached in person if they live in the greater Ann Arbor area, and they will be taught how to place the continuous glucose monitor via a video call with a trained staff member. If the participant already has a continuous glucose monitor as part of their normal care, we will ask to be connected to their data. After wearing the continuous glucose monitor for 14 days, participants will mail the device back to the program staff, (4) report their blood pressure measured on the monitor provided by the study 8 times over 2 weeks, (5) measure their body weight on the scale provided twice within 5 minutes to provide an accurate average, and (6) meet online with a study physician to discuss medications and glucose testing, if they are using insulin or a secretagogue to manage their diabetes. Once all baseline steps have been completed, the program will begin asynchronously for each participant. For details, see the SPIRIT flowchart in Figure 1 for the screening and intervention timeline.

### Sample Size

To estimate the number of subjects needed for the outcome of HbA_1c_, we note that the Diabetes Prevention Program showed a 0.1% decrease in HbA_1c_ with an SD of the difference approximately equal to 0.1% [33]. This decrease in HbA_1c_ was deemed clinically significant because it led to a clear reduction in type 2 diabetes incidence compared to the control. The DPP’s SD estimate of 0.1% is consistent with 2 studies of very low–carbohydrate diets in adults with prediabetes [34,35]. Thus, we used 0.1% as the SD in our sample size calculation. For a 2-sided, paired *t* test to detect a clinically meaningful difference of 0.1% and a SD of 0.1%, we have more than 90% power with 84 retained participants.

### Progression

We have denoted the progression criteria that would inform a decision to proceed to a full-scale trial. We would proceed if the 95% CIs for our measures of acceptability, feasibility, as well as for our health outcome of HbA_1c,_ all meet the following benchmarks, respectively: a mean rating of at least 3.0 on the 0-6 satisfaction scale, at least 40% attendance of assigned sessions, and a decrease of at least 0.1% SD.

### Recruitment

To reach our target sample size, we will advertise on social media to reach participants across the United States. We will hang fliers in community centers in various locations in the United States. We will also search the medical records of Michigan Medicine for potentially eligible participants. Each year of the study, we will initially contact the pool of potentially eligible participants by sending them a letter and flyer using a Health Insurance Portability and Accountability Act (HIPAA)–compliant company describing the study, which will refer them to a study webpage and an initial online screening questionnaire. All recruitment materials will be written at no higher than a fifth-grade reading level. We may follow up with a phone call.

### Data Collection and Management

#### Plans for Assessment and Collection of Outcomes

Assessment data will be reviewed for accuracy and completion by study staff. Loss to follow-up and incomplete data will be recorded.

#### Plans to Promote Participant Retention and Complete Follow-Up

Participants will be paid US $100 for completing the 4-month assessments and will be able to keep any of the intervention-related items we send them, including a body weight scale, a blood pressure cuff, and a glucometer. We also allow them to choose a few kitchen items, such as a skillet, a baking sheet, or ingredients, to send to them at the beginning of the study.

#### Data Management

Trial data will be collected through online questionnaires via Qualtrics. Labcorp will send laboratory data via electronic files to be added to the study database. There will be a regular review of cases during the enrollment process to ensure proper eligibility. All study data will be stored in REDCap (Research Electronic Data Capture; Vanderbilt University), a cloud-based HIPAA-compliant database. REDCap allows for specified ranges and automatic calculations to reduce errors. Data will be cleaned by the research team upon completion of data collection.

#### Plans for Collection, Laboratory Evaluation, and Storage of Biological Specimens for Genetic or Molecular Analysis in This Trial or Future Use

Participants will have blood samples drawn at baseline and 4 months later. All blood samples will be drawn, analyzed, and then destroyed by Labcorp, excluding the rare exception where participants will be mailed a DTI Laboratories kit for their 4-month HbA_1c_ measurement.

### Statistical Analysis

A biostatistician will perform all outcome statistical analyses. An intention-to-treat analysis will be performed on all participants. A per-protocol analysis will also be used on participants who had high compliance with the very low–carbohydrate breakfast intervention. We defined high compliance as breakfasts that have 20% or less of their calories from net carbohydrates.

Descriptive statistics will be computed for all outcome measures. Continuous variables will be reported using means, medians, IQRs, and SDs based on the distribution. Categorical variables will be presented using frequencies and percentages.

#### Aim 1: Assess Acceptability and Feasibility

##### Overview

For this aim, we will explore how measures of acceptability and feasibility change from baseline to the end of the study using a 2-tailed paired *t* test for continuous measures and a test of proportions for dichotomous measures. The magnitude and direction of the effect will be reported. Graphical summaries and Q-Q plots will assess the appropriateness of the normality assumption for the *t* tests. If normality is not indicated, we will take remedial measures such as transformations or nonparametric analyses.

##### Power Aim 1

For the primary outcome measure of acceptability, we have denoted a mean rating of 4.0 on the 0-6 satisfaction scale as our *a priori* benchmark for acceptability, above the midpoint of the scale, with a meaningful difference of 5.0, one point above. We based our estimate of variance on the satisfaction rating from our most recent 2 trials of adults using our 16-week program (SD=1.3 and SD=0.7). Our proposed trial has >90% power to detect this level of satisfaction, using the conservative values of 1.3 for the SD, using a 2-tailed *t* test at an ⍺ level of 5%.

Thus, we have at least 85% power for both primary hypotheses of Aim 1.

#### Aim 2: Assess Changes in Health and Psychological Outcomes

For continuous health and psychological measures, we will explore the change from baseline to the end of the study for each outcome, using a 2-tailed paired *t* test, with a significance level of 0.05 and 95% CIs. As in Aim 1, we will test the assumption of normality and remediate as necessary. For the dichotomous variable measuring achievement of glycemic control, we will report descriptive statistics in comparison to other interventions, such as the Diabetes Prevention Program [36].

#### Interim Analyses

There are no planned interim analyses or stopping rules for this trial. All intervention-related serious adverse events will be reviewed by the Data and Safety Monitoring Board to determine if the study should be stopped.

#### Methods for Additional Analyses (eg, Subgroup Analysis)

We will analyze whether baseline characteristics modify the benefits of the low-carbohydrate breakfast diet on HbA_1c_. We will explore whether there seem to be differences in the magnitude of HbA_1c_ changes from baseline to 4 months later across 3 predefined subgroups: levels of obesity, levels of insulin resistance, and women versus men.  Linear mixed models similar to those described above will be used, with the addition of candidate moderators (each in separate models), and interactions between the moderator, intervention arm, and time. These models will be used to estimate the change in the outcome within subgroups and differences in change between subgroups. The focus of these exploratory analyses will be the magnitude and direction of change within and between subgroups. We will also report the statistical test on the interaction term, which represents an overall test of the moderation effect.

#### Methods in Analysis to Handle Protocol Nonadherence and Any Statistical Methods to Handle Missing Data

To assess the impact of missing data on our outcomes, baseline characteristics of participants will be compared with and without missing data. Primary outcome analyses will use random-intercept-random-slope mixed effects models to approximate changes from the start to the end of the study, 4 months later. Mixed effects models using maximum likelihood estimation allow an appropriate assessment of repeated measures despite the effects of missing data.

#### Plans to Give Access to the Full Protocol and Participant-Level Data

After the trial’s prespecified outcomes have been published, a deidentified dataset will be available to other investigators by request. For access to the dataset, the request must align with institutional review board protocols, and the trial steering committee must agree to the request.

### Oversight and Monitoring

#### Composition of the Coordinating Center and Trial Steering Committee

The primary decision-making body of this study is the investigative team comprising the principal investigator (PI; LRS) and the coinvestigators. The PI is accountable for the overall management of the study. She directs the operations of the study, reviews questions or problems that arise from the study between team meetings, and presents issues to the research team for decision. The PI acts as the intermediary with the funding organization, handling tasks such as submitting annual reports, overseeing fiscal and administrative operations, and managing the coordination and implementation of the study.

The trial steering committee will consist of the PI (LRS), trial coordinator (KR), and study physician (SK). Weekly meetings with the study team will be conducted to review study implementation and discuss adverse events. Additionally, monthly meetings will be held with other study investigators and staff to address any overarching study issues and assess trial progress. Ongoing communication through group email will facilitate discussions related to enrollment inquiries and address issues like recommending medication changes for participants.

The day-to-day operations of the study, encompassing recruitment, data collection, and intervention processes, are overseen by project coordinators and research assistants. Their responsibilities extend to coordinating institutional review board revisions, managing data, monitoring reports, and documenting the completion of necessary trainings. Staff members are tasked with participant recruitment and screening, securing informed consent, and scheduling and conducting follow-up assessments. The lead project manager supervises the development of the study’s data tracking system and surveys.

#### Composition of the Data Monitoring Committee, its Role, and Reporting Structure

A data safety monitoring board, composed of 3 individuals, has been established. Biannual board meetings will be conducted through video conference to assess recruitment, retention, and safety reports, as well as preliminary results.

#### Adverse Event Reporting and Harms

In the event of a serious adverse event with a likely connection to study participation, a special meeting with the data and safety monitoring board will be convened to assess the necessity for any required modifications or potential early termination of the trial. An adverse event is broadly defined as any occurrence that causes or elevates the risk of harm to the participant or others. Serious adverse events encompass instances leading to death, inpatient hospitalization, or extension of existing hospital stay, persistent or significant disability or incapacity, or the emergence of a congenital anomaly or birth defect. The study team meticulously evaluates all potential adverse events reported by participants, considering their relation to the study intervention, expectedness, and severity.

Following a reduced-carbohydrate diet may result in reduced glucose levels. For individuals taking medications like insulin or a secretagogue, this could elevate the risk of hypoglycemia if appropriate medication adjustments are not implemented. Medical adjustments, particularly reductions (or discontinuations at low doses), will be supervised by study physicians. The order of prioritizing reductions or discontinuations typically follows the sequence outlined: (1) insulin, (2) secretagogues, (3) meglitinides, (4) sodium-glucose transport-2 inhibitors, (5) glucagon-like peptide -1 agonists, glucagon-like peptide 1/glucose-dependent insulinotropic polypeptide dual agonists, and dipeptidyl peptidase-4 inhibitors, (5) alpha glucosidase inhibitors, and (6) thiazolidinediones.

Upon enrollment in the study, we will provide participants’ primary care physicians with study information.

The study team will consult the study endocrinologist for potential medication changes if the participants report a low blood glucose level below 90 mg/dL on their weekly survey or if they report their blood glucose as dropping below 110 mg/dL on 2 separate occasions. If the endocrinologist advises medication changes, this will be communicated to the participant. We will fax any changes to the primary care physician.

If participants decide not to accept the medication changes, the study team will also fax the primary care physician our recommendation and the participants’ rejection of the suggestion.

#### Frequency and Plans for Auditing Trial Conduct

The trial will be closely monitored by study investigators and the data and safety monitoring board, convening twice annually. The study team will submit annual progress reports to both the institutional review board and the National Institute of Diabetes and Digestive Kidney Diseases, the study sponsor.

### Dissemination Plans

The trial results will be disseminated through conference presentations, uploaded onto ClinicalTrials.gov (NCT05986097, initially registered on August 2, 2023), and published in peer-reviewed journals. All final peer-reviewed manuscripts will be submitted to the digital archive PubMed Central. Additionally, relevant data will be deposited in suitable public repositories where applicable.

### Ethical Considerations

This trial uses a single institutional review board, with the primary application approved by the University of Michigan Institutional Review Board (HUM00225646; Office for Human Research Protections Institutional Review Board registration number IRB00000244). When communicating important protocol modifications, the study team will request approval from the University of Michigan Institutional Review Board via eResearch and communicate approved changes to the relevant parties via email, phone, or online meetings. We plan to confirm that all groups understand and agree with the proposed changes. All trial participants provide informed, written consent using forms approved by the institutional review board. We will deidentify and code all surveys and forms with a unique participant number. To encourage participant retention, participants will be paid US $100 for completing the 4-month assessments.

## Results

As of October 2025, we have enrolled 119 participants. Recruitment was initiated on August 2, 2023, and the date for completion was October 17, 2025. The results will be published separately.

## Discussion

### Primary Findings

This paper describes the protocol for the Breakfast Study, an acceptability, feasibility, and preliminary effectiveness trial in 115 adults with type 2 diabetes and persistent hyperglycemia. We will assign adults with an HbA_1c_ of 7.0% or higher to our 4-month, digital, small-steps, very low–carbohydrate breakfast-focused program (The Breakfast Study).

This study aims to establish the feasibility and effectiveness of a dietary intervention that only requires changing breakfast to a very low–carbohydrate one. A previously conducted trial reported improvements in glucose control (reduction in antihyperglycemic medications and HbA_1c_), with a reduced carbohydrate (moderate carbohydrate), higher calorie breakfast, when compared to a higher carbohydrate breakfast of a smaller caloric portion [11]. Another trial that compared a very low- to a high-carbohydrate breakfast for people with type 2 diabetes showed a reduction in hyperglycemia, postprandially, and a lower perception of hunger predinner [12]. This trial is aimed at addressing important knowledge gaps that have been noted by the American Diabetes Association and other experts in making clinical guidelines for the nutritional management of type 2 diabetes.

### Limitations

There are several limitations in this trial. First, because it aims to establish acceptability, feasibility, and preliminary effectiveness, it is not a randomized trial; all participants will be in the intervention group. There are limitations inherent in the single-arm design of this study, as it may not fully address key feasibility aspects required to inform a future randomized controlled intervention. Specifically, we will need to address issues such as participant satisfaction with randomization and adherence to the future control arm of the study. Additionally, the absence of a control group may result in inflated effect estimates. However, the single-arm design was chosen as a pragmatic first step to explore initial feasibility and acceptability, refine the intervention, and identify potential barriers to implementation of the intervention. If this trial is successful, the intent would be to conduct a larger randomized controlled intervention of a very low–carbohydrate breakfast diet intervention.

The trial is also limited in the method of measuring dietary adherence, continually asking participants throughout the trial about their diet, but there is no way of validating the information that is received. The design of the trial is completely online and remote (with rare exceptions based on participant need), thus participants must be technologically literate to participate. The study team is trying to mitigate this limitation by mailing physical copies of the curriculum and recipe book, as well as participant lab forms for their blood work, if requested. This trial also relies on the participants having a physical address so that materials can be mailed to them throughout the study. This is not a meal replacement study, so participants must have the financial means and the mental capacity to adhere to our dietary recommendations.

### Conclusion

To our knowledge, the Breakfast Study is the first of its kind assessing the feasibility and efficacy of changing to a very low–carbohydrate breakfast in patients with type 2 diabetes and persistent hyperglycemia. If such an intervention is feasible and efficacious at controlling diabetes, it would be an important intervention to study more, as it is much easier to implement than a completely ketogenic diet.

## Appendix

Multimedia Appendix 1 SPIRIT checklist.

Multimedia Appendix 2 Peer-review report from: ZRG1 RPHB-C (90) - Center for Scientific Review Special Emphasis Panel, Risk Prevention and Lifestyle Change, Behavioral Medicine, Psychosocial Development, and Behavioral Health Outcomes (National Institutes of Health, USA).

## Abbreviations

CONSORT: Consolidated Standards of Reporting Trials
HbA_1c_: glycosylated hemoglobin
HIPAA: Health Insurance Portability and Accountability Act
PI: principal investigator
REDCap: Research Electronic Data Capture
SPIRIT: Standard Protocol Items: Recommendations for Interventional Trials

## Data Availability

Deidentified participant data will be available upon reasonable request to the corresponding author and pending institutional review board approval.
