# Supplementary material for: Very Low–Carbohydrate Breakfast Intervention for Adults with Type 2 Diabetes and Persistent Hyperglycemia: Protocol for a Digital, Nonrandomized Pre-Post Study
Source: JMIR Res Protoc. 2026 Jan 28;15:e81041. doi: 10.2196/81041 (PMC12895149; doi:10.2196/81041)
Supplement: Multimedia Appendix 2 [file resprot_v15i1e81041_app2.pdf]

COMMITTEE BUDGET RECOMMENDATIONS
NEW INVESTIGATOR
PROTECTION OF HUMAN SUBJECTS UNACCEPTABLE

RESUME AND SUMMARY OF DISCUSSION: In this application, the investigators propose to improve health outcomes for adults with poorly controlled type 2 diabetes by testing a potentially effective breakfast-focused self-management intervention. The review committee considered the scientific foundation of the study well supported by scientific literature and by investigators prior work. They thought the study to be significant as there is a critical need to provide additional effective options for population-level type 2 diabetes treatment strategies, especially for adults with poorly controlled type 2 diabetes, the proposed study if successful, would help in type 2 diabetes treatment strategies. They considered the investigative team well experienced to conduct the studies, while lacking expertise in the area of psychology. Other noted strengths include the examination of low-carbohydrate breakfast program among adults with uncontrolled type-2 diabetes to be innovative, well justified outcomes, appropriate statistical approaches, well justified sample sizes, use of biological variables, and appropriate recruitment and retention strategies. However, concerns were noted in the rigor of the approach. These include limited details on the consideration of the race/ethnicity as potential confounder, limited details on the recruitment of minority population, lack of a clear justification as to why an experimental design is not considered, and selection bias in the proposed recruitment strategy. Also, reviewers noted the inclusion criteria with glycated hemoglobin levels to be narrow. Following discussion, the review committee agreed the study would have high impact in improving the health outcomes for adults with poorly controlled type 2 diabetes.

DESCRIPTION (provided by applicant): More than 15% of U.S. adults with type 2 diabetes have poorly controlled blood sugar, here defined as a glycated hemoglobin (HbA1c) level of 9.0% or higher. These adults have an elevated health risk of a variety of outcomes, including amputation and mortality from cardiovascular disease and from all causes. Nutrition- focused interventions can be effective for improving glycemic control, reducing anti-hyperglycemic medications, and reducing body weight, all of which are critical outcomes for adults with type 2 diabetes. However, typical nutrition-focused interventions can be burdensome, often requiring complex instructions and a complete overhaul of one's diet. Additionally, adults with poorly controlled type 2 diabetes are more likely to have low literacy levels, which can be a barrier for adherence to complex interventions. Therefore, an effective intervention for adults with poorly controlled diabetes who may have lower health literacy levels is necessary to reduce both HbA1c levels and anti-hyperglycemic medications. Carbohydrate intake has the strongest impact on post-prandial glycemia of any dietary factor, and a very low-carbohydrate diet—due to its ability to improve glycemic control—is now recommended by the American Diabetes Association (ADA) for the treatment of type 2 diabetes. We hypothesize that some of the benefits of a very low-carbohydrate diet may be available to individuals who change only their breakfasts to be very low-carbohydrate, rather than modifying their entire diet. Thus, we propose an acceptability, feasibility, preliminary effectiveness trial of a 4-month, online, small- steps, low-literacy, very low-carbohydrate breakfast-focused program in 120 adults with poorly controlled type 2 diabetes. We will measure acceptability and feasibility, plus critical efficacy outcomes, such as changes in HbA1c, anti-hyperglycemic medications, glycemic variability, body weight, blood pressure, and lipids. We will also test whether factors such as sex, health literacy level, and baseline insulin resistance significantly moderate the impact of the intervention on change in HbA1c and change in anti-hyperglycemic medications. These moderation analyses will contribute to a nascent research approach for personalized nutrition, which responds to Objective 2-5 of the NIH's May 2020 Strategic Plan for NIH Nutrition Research. This submission is in response to PAS-20-160, Small R01s for Clinical Trials

Targeting Diseases within the Mission of NIDDK, which does not require preliminary data. If results are promising, our goal is to conduct a follow-up, powered, longer randomized trial of this approach. As the prevalence of type 2 diabetes and the understanding of personalized interventions continue to increase, there is a critical need to provide additional effective options for population-level type 2 diabetes treatment strategies, especially for adults with poorly controlled type 2 diabetes.

PUBLIC HEALTH RELEVANCE: This work aims to help improve health outcomes for adults with poorly controlled type 2 diabetes by testing a potentially effective breakfast-focused self-management intervention. We hypothesize that this will be a feasible and effective option.

CRITIQUE 1

Significance: 2
Investigator(s): 3
Innovation: 3
Approach: 5
Environment: 2

Overall Impact: This study is designed to test the acceptability, feasibility and preliminary effectiveness of a digital small step very low-carbohydrate breakfast trial in 120 adults with poorly controlled type 2 diabetes. Additionally, subgroups by age, sex, health literacy level, emotional eating and baseline insulin resistance who will benefit from the intervention will be determined and results will be used for a future randomized trial. This study addresses a significant problem and is headed by a well-rounded team. Moreover, its novel approach of addressing a single meal is attractive to participants with low literacy. Overall, its strengths outweigh minor weaknesses.

1. Significance

Strengths

- Focus on a major health issue - poorly controlled diabetes is a major contributor to disability and death
- Lifestyles change that has potential to reduce need for anti-diabetic medications

Weaknesses

- None noted by reviewer.

2. Investigator(s)

Strengths

- PI (Dr. Saslow) has experience in diet and PA interventions; includes nutrition scientist (Dr. Leung) and endocrinologist (Dr. Kim) and there is also a biostatistician (Dr. Isaman)

Weaknesses

- Would benefit from involvement with psychologist as emotional eating is being targeted

3. Innovation

Strengths

- Redesigning breakfast so that it is both appetizing and likely to reduce glycemia
- Introducing non-traditional protein sources (e.g., almond flour) for making bread
- On-line intervention and daily motivational text messages to help participants stay on track

Weaknesses

- Focus on one meal of the day

4. Approach

Strengths

- Focus on diet- a very low carbohydrate breakfast- as carbohydrates have the strongest impact on post-prandial glycemia
- Focus on a low literacy strategy by just focusing on breakfast as opposed to the whole day
- On-line small steps intervention
- Outcomes include changes in psychological outcomes, HbA1c, anti-hyperglycemic medications, glycemic variability, body weight and blood pressure and lipids
- Assesses impact of sex as a biological variable
- Safety issues addressed by having an initial session on medications to review need for changes (e.g., if on short acting insulin for breakfast- this would be discontinued)
- First six weeks tight monitoring of glucose levels to ensure participants are not over-medicated

Weaknesses

- Does not address breakfast skippers
- Requires major changes to breakfast that requires cooking (e.g., omelets) hard to accommodate breakfast on the go

5. Environment

Strengths

- Excellent

Weaknesses

- None noted by reviewer.

Study Timeline:

Strengths

- Overall appropriate

Weaknesses

- May need more time to retain 120 participants in this 4 month study

Protections for Human Subjects:

Acceptable Risks and/or Adequate Protections

Data and Safety Monitoring Plan (Applicable for Clinical Trials Only):
Acceptable

Inclusion Plans:

- Sex/Gender: Distribution justified scientifically
- Race/Ethnicity: Distribution justified scientifically
- For NIH-Defined Phase III trials, Plans for valid design and analysis: Scientifically acceptable
- Inclusion/Exclusion Based on Age: Distribution justified scientifically

Vertebrate Animals:
Not Applicable (No Vertebrate Animals)

Biohazards:
Not Applicable (No Biohazards)

Resource Sharing Plans:
Acceptable

- Discussed in dissemination plan

Budget and Period of Support:
Recommend as Requested

CRITIQUE 2

Significance: 2
Investigator(s): 1
Innovation: 1
Approach: 2
Environment: 1

Overall Impact: This R01 application in response to a funding opportunity announcement for small pilot and feasibility trials describes a pilot trial with pre-post design of a low carbohydrate breakfast diet program intervention for adults with uncontrolled type 2 diabetes. The application is strengthened by exceptional investigator team is led by a PI who is a productive New Investigator and K01 recipient complemented by an accomplished and experienced study team with a strong track record of collaboration and successful projects. Other score driving strengths include the very high significance, supported by rigorous evidence that is appropriately evaluated in the application, very high innovation of the intervention itself and the focus of the study on important gaps in knowledge, and an ideal research environment for the proposed work. The proposed approach is rigorous with many strengths including strong rationale for the intervention components, that have largely been developed and are in use, and the outcome measures. Some moderate weaknesses in the approach were also noted.

Although a pre-post design brings sufficient rigor to a pilot trial, it was not clear why an experimental design was not proposed. Also, although the recruitment plan seems appropriate to achieve the total sample size targets, more attention to the recruitment and retention of minority participants would add to the ability of the study to generate rigorous and unbiased results. Taken together, the expected overall impact of the proposed work is high.

1. Significance

Strengths

- The public health significance of uncontrolled type 2 diabetes is large
- T2DM interventions that focus solely on medication intensification may hinder adherence and self-management, have mixed impact on glycemic control and limited to no impact on weight change
- Rigor of evidence surrounding medication intensification regimens was appropriately evaluated, and gaps in knowledge around limited reporting on medication changes and weight changes were identified
- Evidence for benefits of nutrition based interventions was provided, included reference to findings from an ADA Nutrition Review Committee
- Rigorous evidence for the plausibility of small step dietary changes, namely low carbohydrate breakfast, was provided with a strong argument for the significance of the proposed research questions

Weaknesses

- Rigor of the studies contributing to knowledge on nutrition based intervention was not explicitly evaluated, relying instead on evidence synthesis
- Discussion of gaps in knowledge around barriers to successful nutritional interventions was limited

2. Investigator(s)

Strengths

- The PI is an accomplished nursing researcher and K01 recipient with numerous contributions to the field, established collaborations with other members of the study team, and experience
- Collectively, the team has expertise in all requisite areas for the project and an established track record leading successful federally funded studies and clinical trials.
- Dr. Isaman provides the necessary statistical expertise

Weaknesses

- None noted by reviewer.

3. Innovation

Strengths

- Examining a low-carbohydrate breakfast program among adults with uncontrolled T2DM is innovative

- Examining medication changes is often overlooked in nutrition and other diabetes self-management intervention studies, and is also somewhat innovative

Weaknesses

- None noted by reviewer.

4. Approach

Strengths

- Although preliminary data is not required for this funding mechanism, the investigators' prior work designing, and testing intervention components supports the appropriateness and feasibility of the study materials (major)
- Pre-post design is acceptable for a pilot trial, and will allow rigorous assessment of within-person changes (moderate)
- Selection of outcome measures are appropriate and well-justified (moderate)
- Recruitment and retention strategy seems appropriate to achieve the required total sample size for the project, and will be evaluated as part of the feasibility analysis (moderate)

Weaknesses

- No clear justification for why an RCT or other experimental design is not being proposed, as this would add additional rigor and the ability to estimate preliminary intervention effect sizes (moderate)
- The plan to recruit minority participants is somewhat underdeveloped, which may introduce bias into the research plan (moderate)
- The statistical power and sample size requirements for the Aim 3 moderation analysis was not discussed, but this is only a minor weakness as the analysis is largely exploratory (minor)

5. Environment

Strengths

- The applicant's institution has all the necessary research infrastructure and resources to support this work
- The clinical environment appears to be suitable to identify a sufficient number of eligible patients for the mail plus online recruitment strategy

Weaknesses

- None noted by reviewer.

Study Timeline:

Strengths

- An appropriate and feasible timeline was provided

Weaknesses

- None noted by reviewer.

Protections for Human Subjects:

Unacceptable Risks and/or Inadequate Protections

- Although the investigators give appropriate attention to hyperglycemia risk in the Protection for Human Subjects plan, it is not clear how medication changes prescribed by the study physician will be coordinated with the participant's personal physician.

Data and Safety Monitoring Plan (Applicable for Clinical Trials Only):

Acceptable

- A DSMB will be established with roles and timeline for reviews and reporting clearly described

Inclusion Plans:

- Sex/Gender: Distribution justified scientifically
- Race/Ethnicity: Distribution justified scientifically
- For NIH-Defined Phase III trials, Plans for valid design and analysis: Not applicable
- Inclusion/Exclusion Based on Age: Distribution justified scientifically
- Inclusion Enrollment plan based on national demographics of individuals with uncontrolled diabetes and appropriately represents minority participants and women. An acceptable scientific justification for limiting enrollment to adults under age 80 was provided, even though some older adults will not be included.

Vertebrate Animals:

Not Applicable (No Vertebrate Animals)

Biohazards:

Not Applicable (No Biohazards)

Resource Sharing Plans:

Not Applicable (No Relevant Resources)

Budget and Period of Support:

Budget Modifications Recommended (in amount/time)

Recommended budget modifications or possible overlap identified:

- The budget is very lean. Increased time for project coordinators could add to the feasibility and impact of the study

CRITIQUE 3

Significance: 3
Investigator(s): 1
Innovation: 3

Approach: 4
Environment: 1

Overall Impact: In response to PAS-20-160, *Small R01s for Clinical Trials Targeting Diseases within the Mission of NIDDK,* the investigative team proposes to examine the acceptability, feasibility, and preliminary effectiveness of the 4-month "Breakfast Trial" among 120 adults with poorly controlled type 2 diabetes (T2D) defined by a HbA1c of ≥9.0%. This is a Phase II clinical trial where all study participants will receive the low-carbohydrate breakfast focused intervention with the overall goals to assess 1) feasibility and acceptability, 2) changes in health and psychological outcomes, and 3) factors of individuals that may benefit from the proposed intervention. T2D results in considerable burden in terms of prevalence and associated costs and this burden is often higher among those with poorly controlled T2D. As proposed, the overall impact is expected to be medium/high because it addresses a need to develop and evaluate nutrition-based interventions rather than solely rely on medication intensification, which may result in poorer outcomes. Low or very low carbohydrates have been shown to be effective for improving diabetes management. This proposed intervention addresses an important limitation through modification of the breakfast meal, rather than all daily meals which may be a more feasible and acceptable approach. The investigative team and environments are strong and leverage prior work to support this intervention. However, in my review, I noted a few minor/moderate weaknesses primarily regarding significance and approach that reduced my overall enthusiasm. First, the proposed intervention is designed to target adults with poorly controlled T2D who are also low literacy, which is further operationalized as low health literacy. However, recruitment methods and eligibility criteria may not yield an adequate sample to evaluate this or explore for potential moderation by health literacy status. Second, race/ethnicity may also be an important potential moderator to examine; particularly given known race/ethnic disparities re: diabetes management. Third, selection bias could result from the proposed recruitment strategy to 1) recruit from medical records and 2) require participants to make initial contact. Finally, weekly session topics for the intervention were more general and not specific to breakfast. Further contrasting the breakfast intervention against the investigative team's prior intervention studies would provide additional rationale for the importance of this study. Despite these weaknesses, the potential impact remains medium to high.

1. Significance

Strengths

- T2D is a highly prevalent disease and one of the most expensive health conditions in the U.S. If the current trajectory continues, nearly 1 and 3 U.S. adults will be diagnosed with T2D by 2050.
- Approximately 15% of Americans have poorly controlled T2D (HbA1c of ≥9.0%) which increases subsequent risk of amputation and premature death (all-causes and CVD related).
- Medication intensification, a standard approach for poorly controlled T2D, is potentially problematic given it adds complications to self-management routines and can lead to overmedication and poor outcomes (e.g., hypoglycemia). Further, the overall effectiveness of this approach is unclear given lack of reporting on medication changes.
- Digital, remote based programs are often used to deliver interventions focused on self-management programs. Again, the overall effectiveness of these programs are unclear given lack of reporting on medication changes and weight loss.
- Prior research has supported the effectiveness of nutrition-based programs to improve management of T2D and low- or very low carbohydrate diets are particularly effective. However, these types of diets are complex and time-consuming which could lead to non-adherence.

- PI Saslow has a funded K01 that is designed to test an online, very low carbohydrate diet among adults diagnosed with T2D taking metformin or no anti-hyperglycemic methods, which has gleaned promising findings (qualitative and quantitative) in support of this proposed study.

Weaknesses

- Intervention to be tested is designed to be a small-steps, low (health) literacy very low carbohydrate breakfast-focused program. Health literacy will be assessed using a 4-item survey and tested as a potential moderating variable. Yet, the eligibility criteria may not result in sufficient numbers to adequately test this.
- Racial/ethnic disparities exist; however, Aim 3 does not include race/ethnicity as a potential confounder.

2. Investigator(s)

Strengths

- Strong investigative team with a productive collaborative history and the necessary complementary and integrative expertise to successfully accomplish the proposed study aims.
- PI Saslow served as a lead reviewer of the ADA's Nutrition Review Committee and has an ongoing K01 funded study designed to optimize an online intervention to improve blood glucose control of overweight individuals with T2D. PI Saslow is also PI of two recently funded R01s testing nutritional interventions for individuals with T2D. She has the expertise and experience to successfully lead this proposed study.

Weaknesses

- None noted by reviewer.

3. Innovation

Strengths

- If successful, the proposed intervention focused on breakfast may be a promising approach to improve diabetes management for those with poorly controlled T2D. In clinical practice, this approach would serve as an incremental step towards transition to a very low carbohydrate diet, overall.

Weaknesses

- Minor/Moderate. While the approach to examine potential differences in the effectiveness of the intervention by factors is reasonable, the identification of the specific factors under study and rationale of these factors requires further justification.

4. Approach

Strengths

- The proposed intervention is theory-based and developed/adapted from a prior online very low carbohydrate diet developed by the PI and investigative team with data that suggest overall acceptability and compliance.
- The example very low-carbohydrate diet breakfast options do not require a complete overhaul to foods typically consumed for this meal and the investigative team has developed a variety of materials to support/encourage participants to adhere to the intervention.

- Asynchronous format provides more flexibility and is usually more appealing to most adults.
- The retention plan is appropriate; supported by evidence of good retention from a prior 4-month pilot trial.
- The outcomes for each aim and operationalization of these outcomes are clear and well justified.
- Sex as a biological factor addressed in Aim 3, i.e., planned analyses to examine potential effect modification by sex.
- Possible safety concerns re: hypoglycemia were provided and are well justified.
- The statistical approach and targeted sample size are appropriate and well justified.

Weaknesses

- Minor/Moderate. **Recruitment -** Selection bias is a concern given individuals will be recruited based on making initial contact from study in response to online ads and mailing from a medical record based listing.
- Minor. Eligibility Criteria **–** 1) As conceived, the intervention is targeted to adults diagnosed with low (health, only?) literacy – however, to be eligible a participant must be able to read in English. While materials will be written at a 5th grade reading level, this approach may not fully reach the intended population and 2) The fasting blood draw for HbA1c is required to determine study eligibility, however, will be conducted following the collection of other baseline measures (e.g., 24-hour dietary recall).
- Minor. Intervention **–** 1) The weekly session topics are general and not specific to the breakfast meal and 2) Weekly sessions will include links to resources; however, the target sample are those with low (health) literacy. It is unclear the extent to which this component will be used as intended.

5. Environment

Strengths

- The environments at the University of Michigan and University of California, San Francisco are strong and provide the requisite institutional support and resources to the investigative team to accomplish the proposed study aims.

Weaknesses

- None noted by reviewer.

Study Timeline:

Strengths

- While more granular study activities were noted, the timeline supports the completion of planned study activities in the requested 3-year funding period.

Weaknesses

- None noted by reviewer.

Protections for Human Subjects:

Acceptable Risks and/or Adequate Protections

- Well-developed plan to ensure protection of study participants

Data and Safety Monitoring Plan (Applicable for Clinical Trials Only):
Acceptable

- Well-developed plan to convene and engage with a DSMB

Inclusion Plans:

- Sex/Gender: Distribution justified scientifically
- Race/Ethnicity: Distribution justified scientifically
- For NIH-Defined Phase III trials, Plans for valid design and analysis: Not applicable
- Inclusion/Exclusion Based on Age: Distribution justified scientifically
- The proposed intervention is a Phase II trial

Vertebrate Animals:
Not Applicable (No Vertebrate Animals)

Biohazards:
Not Applicable (No Biohazards)

Resource Sharing Plans:
Acceptable

Budget and Period of Support:
Recommend as Requested

THE FOLLOWING SECTIONS WERE PREPARED BY THE SCIENTIFIC REVIEW OFFICER TO SUMMARIZE THE OUTCOME OF DISCUSSIONS OF THE REVIEW COMMITTEE, OR REVIEWERS' WRITTEN CRITIQUES, ON THE FOLLOWING ISSUES:

PROTECTION OF HUMAN SUBJECTS: UNACCEPTABLE
See Reviewer 2 comments

INCLUSION OF WOMEN PLAN: ACCEPTABLE

INCLUSION OF MINORITIES PLAN: ACCEPTABLE

INCLUSION ACROSS THE LIFESPAN: ACCEPTABLE

COMMITTEE BUDGET RECOMMENDATIONS:
The review committee considered the budget to be very lean and recommended an increased time for project coordinators.
